# Frailty syndrome and healthcare for older adults

**DOI:** 10.1590/1516-3180.2024.1424.21052024

**Published:** 2024-07-15

**Affiliations:** I Full Professor, Department of Clinical Medicine, Hospital das Clínicas (HCFMUSP), Faculdade de Medicina, Universidade de São Paulo (USP), São Paulo, SP, Brazil; II Vice-director, School of Medicine, University of São Paulo (USP), São Paulo, SP, Brazil; Full Professor, Department of Cardiopulmonary Diseases, Faculdade de Medicina, Universidade de São Paulo (USP), São Paulo, SP, Brazil; Director of the Scientific Department, Associação Paulista de Medicina (APM), São Paulo, SP, Brazil.

In the early 2000s, Linda Fried et al.,^1^ then affiliated with Johns Hopkins University, described the pathophysiological bases and a phenotype of frailty syndrome in older adults. This research has served as a landmark in the field of geriatrics and has significantly influenced the healthcare provided for older adults across various medical specialties over the years. Despite its ongoing evolution, the concept of frailty syndrome remains crucial and should not be underestimated or overlooked by physicians caring for patients.

Interest in understanding and precisely defining the difference between chronological and biological age predates the work of Fried et al. In the 1980s, for example, frail older adults were already characterized as those dependent on others for daily activities or survival.^2^ Adults of the same age exhibit varying levels of functionality and robustness profiles, indicating diverse rates and trajectories of aging. However, Fried et al. not only defined the processes underlying these differences but also established diagnostic criteria for frailty syndrome at earlier stages.^1^ This enables the early recognition of older patients at higher risk of developing complications due to clinical and surgical interventions, unplanned hospitalization, falls, functional decline, institutionalization, or death. Such criteria facilitate the implementation of interventions to identify the causes of frailty and propose intervention plans to prevent disease progression and the development of dependency, as exemplified by the Frailty Clinic led by the Geriatrics and Gerontology group of the University of Toulouse.^3^

Although frailty syndrome has been defined in various ways, especially by Professor Kenneth Rockwood and his group,^4^ the foundational definition remains consistent: frailty syndrome denotes an age-related state of physiological vulnerability characterized by diminished homeostatic reserve in multiple systems, rendering individuals vulnerable to stressful events. Initially, Fried proposed that the syndrome is caused by immunological and endocrine disorders and sarcopenia. However, extensive scientific literature on frailty has since revealed associations with genetic and epigenetic factors, socioeconomic conditions, life history, chronic diseases, physical activity levels, and other factors interfering with healthy aging.^5^

Several tools have been developed to diagnose frailty syndrome over the years, such as the Fried criteria, which include unintended weight loss, reduced muscle strength, reduced walking speed, fatigue, and low physical activity levels; the Rockwood frailty index; and other practical and concise tools available for use in routine clinical practice.^6^ The optimal approach to diagnosing frailty syndrome and assessing its impact on clinical practice and the prognosis of older adults in primary care and other healthcare levels has been a longstanding debate. More recent studies have highlighted that the effectiveness of diagnostic instruments depends on the context in which they are applied. For example, the Fried phenotype method is an excellent predictor of short-, medium-, and long-term outcomes in primary care settings, where ample time and resources allow for comprehensive testing. In emergency settings, where time is extremely limited and patients may have significant functional impairments (such as reduced mobility hindering gait speed measurement), conducting thorough tests becomes challenging, potentially compromising the information obtained. In such context, instruments such as the Clinical Frailty Scale, also proposed by Rockwood et al., prove to be more informative and practical.^6,7^

Irrespective of the diagnostic tool used, current literature highlights frailty syndrome as a significant prognostic indicator in older adults with heart disease, peripheral vascular disease, cancer, kidney disease, diabetes, hypertension, neurological disorders, and other chronic conditions; in older patients with acute disease admitted to emergency rooms, intensive care units, and other emergency services; and in those undergoing invasive surgical procedures. Therefore, frailty syndrome should be considered in healthcare and complication prevention protocols in all medical practice scenarios.^8^

We have transitioned from relying solely on chronological age to using physiological reserves and functionality as pivotal guides for medical decision-making. The association of this syndrome with acute morbidity indicators proved to be a significant predictor of prognosis during the coronavirus disease 2019 pandemic.^9^ The management of frailty syndrome considerably improves outcomes after femur fracture.^10^ The accurate diagnosis of frailty syndrome facilitates the implementation of effective interventions that reduce the risk of developing various types of surgical complications.^11^ Lastly, addressing the causes of frailty syndrome enables the development of primary care plans aimed at preventing or delaying the onset of dependence.^3^

In conclusion, the degree of the issue in Brazil reflects a rapidly aging population. Brazilian studies indicate that the prevalence of frailty syndrome among community-dwelling older adults ranges from 8% to 10%, with nearly half of older adults classified as pre-frail according to the Fried phenotype criteria.^12^ These rates are significantly higher in long-term care institutions, emergency rooms, hospitals, and outpatient clinics.^13^ Another crucial issue is that the diagnosis of frailty syndrome is dynamic rather than definitive, with multidisciplinary interventions helping pre-frail adults regain non-frail status and frail adults transitioning between states.^14^ This underscores the importance of early detection and multidisciplinary therapeutic interventions to decrease the risk of clinical complications and functional decline.

For these reasons, physicians from all specialties, along with their interdisciplinary teams, should strive to diagnose frailty syndrome in older patients using the most recommended instruments in the literature. This approach enhances planning and the management of interventions, ultimately improving the prognosis and short-, medium-, and long-term outcomes of older patients in routine clinical practice.
